# [Corrigendum] Nucleosome‑binding protein HMGN2 exhibits antitumor activity in human SaO2 and U2‑OS osteosarcoma cell lines

**DOI:** 10.3892/or.2024.8827

**Published:** 2024-10-16

**Authors:** Guojun Liang, Enjie Xu, Chaoqun Yang, Chenglin Zhang, Xiaolong Sheng, Xuhui Zhou

Oncol Rep 33: 1300–1306, 2015; DOI: 10.3892/or.2014.3689

Following the publication of the above article, an interested reader drew to the authors' attention that, in Fig. 5A on p. 1305, the same xenograft tumor image had been selected for a pair of the GFP/U2-OS experiments, where the images from discretely performed experiments were intended to have been shown. Furthermore, upon performing an independent analysis of the data in the Editorial Office, it was noted that the data selected for the ‘SaO2/GFP/24 h’ and ‘SaO2/HMGN2/48 h’ experiments in Fig. 4A, also on p. 1305, were strikingly similar, such that the same data had apparently been chosen to show the results of differently performed experiments.

After having inspected the figures, the authors realized that one of the n=3 experimental results had inadvertently been omitted from Fig. 5A, and the data shown to represent the ‘SaO2/GFP/24 h’ experiment had been chosen incorrectly. The revised and corrected versions of Figs. 4 and 5 are shown on the next page (also note that erroneously written labels have been corrected in Fig. 5C: “Tumor volume” has been replaced by “Tumor weight”). Note that the errors made in terms of the assembly of the data in these figures did not affect the overall conclusions reported in the paper. The authors are grateful to the Editor of *Oncology Reports* for granting them this opportunity to publish a Corrigendum, and apologize to boh the Editor and the readership for any inconvenience caused.

## Figures and Tables

**Figure 4.**
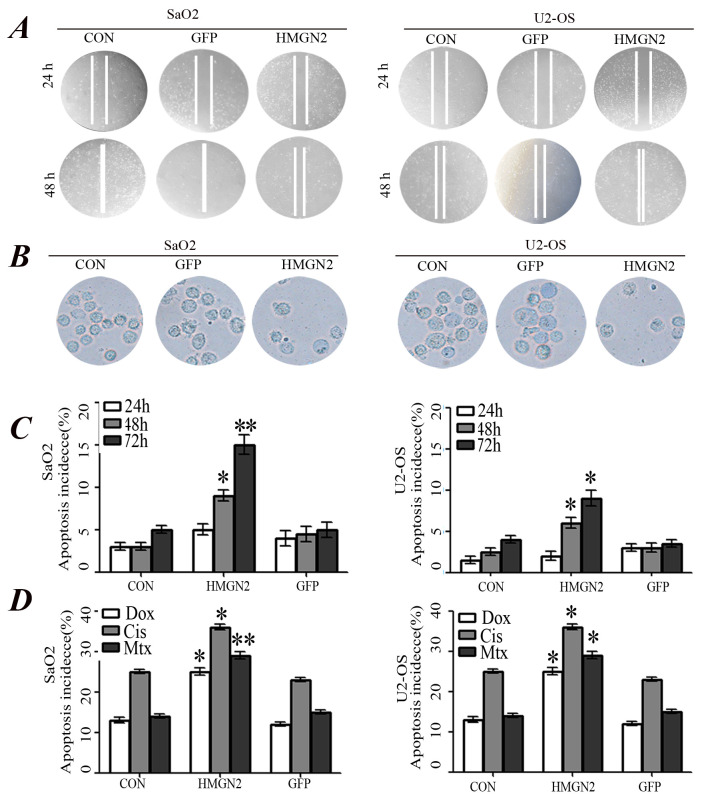
Inhibition of osteosarcoma cell migration and invasiveness by HMGN2. (A) Wound-healing assay showed that the migration capacity of SaO2 and U2-OS cells in the HMGN2 group were markedly lower than those in the GFP and CON groups. (B) Transwell invasion assay for the transmembrane ability of each group of cells. The ability in HMGN2 group was markedly decreased as compared with the GFP and CON groups. (C) The apoptosis of SaO2 and U2-OS cells was analyzed by flow cytometry (Annexin V-FITC/PI) at the end of the incubation period for 24, 48 and 72 h. The apoptosis incidence increased in a time-dependent manner and HMGN2 resulted in a further increase (*P<0.05, **P<0.01), although no difference was found between the GFP and CON groups (P>0.05). (D) Annexin V-FITC/PI staining for apoptosis induced by chemotherapy agents. SaO2/CON, SaO2/GFP, SaO2/HMGN2, U2-OS/CON, U2-OS/GFP, and U2-OS/HMGN2 cells were treated with Dox (0.2 mg/ml), Cis (20 mmol/l), and Mtx (50 mmol/l) for 24 h and the apoptosis incidence was quantified by flow cytometer. Overexpression of HMGN2 resulted in a further increase of apoptosis induced by Dox, Cis and Mtx. (*P<0.05, **P<0.01 vs. CON group).

**Figure 5.**
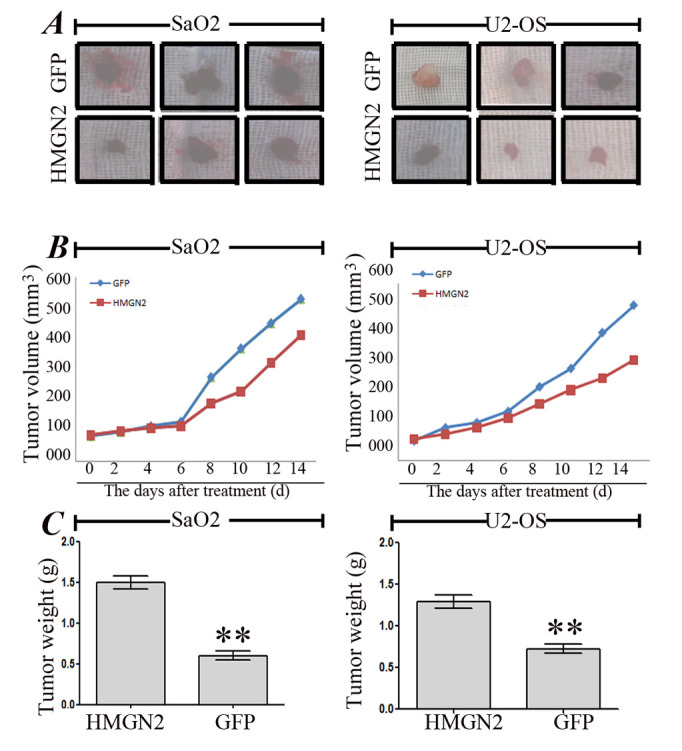
Antitumor effect of HMGN2 in the osteosarcoma SaO2 and U2-OS xenograft models. (A) On day 14, the average volumes of SaO2 and U2-OS xenograft tumors were measured and found to be significantly smaller in HMGN2 group than those in the GFP group. (B) During the whole tumor growth period, the tumor growth activity was measured, showing that the tumors treated with HMGN2 lentivirus grew substantially slower than the GFP group. (C) On day 14, the average weights of SaO2 and U2-OS xenograft tumors were measured and found to be significantly lighter in HMGN2 group than those in the GFP group (**P<0.01).

